# Use of Nanoscale Zero-Valent Iron for Remediation of Clayey Soil Contaminated with Hexavalent Chromium: Batch and Column Tests

**DOI:** 10.3390/ijerph17031001

**Published:** 2020-02-05

**Authors:** Cleomar Reginatto, Iziquiel Cecchin, Karla Salvagni Heineck, Antonio Thomé, Krishna R. Reddy

**Affiliations:** 1 Graduate Program of Civil and Environmental Engineering, University of Passo Fundo, BR 285, km 292, Campus I, Passo Fundo, RS 99001-970, Brazil; thome@upf.br; 2 Environmental Engineering Undergraduate Course, University of Passo Fundo, BR 285, km 292, Campus I, Passo Fundo, RS 99001-970, Brazil; iziquielc@gmail.com; 3 Graduate Program of Civil Engineering, Federal University of Rio Grande do Sul, Av. Osvaldo Aranha, 99 Porto Alegre, RS 90035-190, Brazil; karla@ppgec.ufrgs.br; 4 Department of Civil and Materials Engineering, University of Illinois at Chicago, 842 West Taylor Street, Chicago, IL 60607, USA; kreddy@uic.edu

**Keywords:** soil remediation, nanotechnology, nanoscale zero-valent iron-(nZVI), hexavalent chromium-Cr(VI)

## Abstract

This study investigated the reduction of hexavalent chromium (Cr(VI)) in a clayey residual soil using nanoscale zero-valent iron (nZVI). Five different ratios between nZVI and Cr(VI) were tested in batch tests (1000/11; 1000/23; 1000/35; 1000/70, and 1000/140 mg/mg) with the soil. With the selected proportion resulting best efficiency, the column tests were conducted, with molded specimens of 5 cm in diameter and 5 cm in height, with different nZVI injection pressures (10, 30, and 100 kPa). The soil was contaminated with 800 mg/kg of Cr(VI). The Cr(VI) and Cr(III) analyses were performed following the USEPA 3060A and USEPA 7196A standards. The results show that the reduction of Cr(VI) is dependent on the ratio between nZVI and Cr(VI), reaching 98% of efficiency. In column tests, the pressure of 30 kPa was the most efficient. As pressure increased, contaminant leaching increased. The permeability decreased over time due to the gradual increase in filtration and formation of oxyhydroxides, limiting nZVI mobility. Overall, nZVI is efficient for soil remediation with Cr(VI), but the injection process can spread the contaminated if not properly controlled during in situ application.

## 1. Introduction

Clay soils possess characteristics for the accumulation of contaminants, such as heavy metals and persistent organic pollutants, but their characteristics may also limit the mobility of decontaminating agents during the remediation implementation. Contaminated sites have received great attention among environmental agencies due to their impacts on public health and the environment. Many of the polluted sites exist in urban and industrialized areas, hence their remediation is becoming important and urgent [1,2,3]. Globally there are more than 5 million sites contaminated with metals, generating over 20 million hectares to be remedied [4].

Among the various contaminants from industrial processes, residues containing hexavalent chromium-Cr(VI) are among the most toxic [4,5,6]. In soils, chromium can exist in two different redox states: the immobile trivalent form, Cr (III); and more mobile hexavalent form, Cr(VI) as chromate (CrO_4_^2−^) or dichromate (Cr_2_O_7_^2−^). Cr(III) is a low toxicity nutrient for plant growth, while Cr(VI) is a dangerous, mutagenic and carcinogenic species [7,8,9]. The United States Environmental Protection Agency has declared Cr(VI) as one of the 17 chemicals most harmful to human health [9].

Technologies that make use of nanoscale materials for the remediation of contaminated areas have rapidly developed in recent years, mainly in North America and Europe [10,11]. Among the several types of nanoparticles, the most used for remediation of chromium-contaminated soils was nanoscale zero-valent iron, nZVI [8,12,13,14,15,16,17,18,19]. Moreover, nZVI has the advantage of low toxicity and lower production costs compared to other types of metallic nanoparticles [20,21,22,23,24].

The transport of nZVI in soil differs from the behavior of diluted substances in aqueous solution. In water, the nZVI is better distributed and has easier contact with the contaminant. In the soil nanoparticle, mobility can be limited by mechanical filtration of particles by soil, hydrophilic and hydrophobic interactions, and heterogeneity or interaction with soil [25,26,27]. In order to reduce filtration, several strategies have been adopted, with the most common being the addition of particle coating products, making them more stable, thus becoming more mobile in the medium [28,29,30,31,32].

In recent research for soil contaminated with hexavalent chromium, new means of remediation with the use of nZVI have been studied. However, practically all studies have been for coarse soils and only on a batch scale without a column or field injection process in contaminated soil [3,4,14,15,17,18,33,34].

Particularly for residual clay soils, mainly characterized by low permeability and large particle surface area, the mobility and interaction of the reducing agent with the contaminant needs to be better understood. In this sense, laboratory tests with contaminated soil, evaluating the relationship between the contaminant and nZVI, as well as their movement in the soil, are necessary for subsequent application in in situ remediation processes. Thus, the objective of this work was to optimize and evaluate the remediation of a Cr(VI) contaminated clay soil using nZVI.

## 2. Materials and Methods

### 2.1. Soil

The research was conducted in the Laboratory of Environmental Geotechnics in the Centre of Technology (CETEC) at the University of Passo Fundo (UPF) in southern Brazil. Soil samples of clay soil were collected at a depth of 1.2 m (B horizon) from an open trench in the experimental geotechnics field on the UPF campus. The soil was derived from basaltic rock, and its main physical–chemical and geotechnical characteristics are presented in Table 1.

The pedological classification of the soil is Oxisol, and the geotechnical classification by Unified Soil Classification System (USCS) is clay with high plasticity (CH). The soil is acidic with high clay content, low organic matter content, and low cation exchange capacity (CEC), which is typical of soils with a high content of the mineral kaolinite [35].

### 2.2. Batch Tests

The batch experiments were conducted to evaluate the influence of the relationship between the reducing agent (nZVI) and the contaminant (Cr(VI)), in order to find the best ratio between them for better soil remediation efficiency to meet the allowable limit per the Brazilian Soil Quality Law applicable to industrial sites [36].

These tests were performed with the soil, assuring greater homogenization of the contaminant and reducing agent, because the reduction occurs by a contact reaction between them. The tested ratios of nZVI to Cr(VI) were: 1000 mg nZVI for each 11 mg chromium; 1000 mg nZVI for every 23 mg of chromium; 1000 mg nZVI for every 35 mg of chromium; 1000 mg nZVI for every 70 mg of chromium; and 1000 mg nZVI for every 140 mg of chromium. These ratios resulted in corresponding dimensionless ratios of 90.9, 43.5, 28.6, 14.3, and 7.1. 

All tests were performed with 100 g of soil, with natural moisture of 34%. All batch tests were performed in triplicate for reproducibility of the results. After soil contamination and the addition of the nZVI suspension, the mixture was manually homogenized and after 24 h of contact, the concentrations of Cr(VI) in the soil were analyzed.

### 2.3. Column Tests

The column tests were carried out in a flexible-wall column assembled according to ASTM D4874 [37]. The equipment had the ability to test three specimens simultaneously and more details of the equipment can be found in Reginatto et al. [38]. The equipment allows one to evaluate the hydraulic conductivity throughout the test, and the interactions between the contaminant and the reducing agent with leachable contaminant and the possible elution and filtration of nZVI. 

The soil specimens were tested under three different nZVI injection pressures: 10 kPa, 30 kPa, and 100 kPa. The specimens were confined in the column, under a constant confining pressure of 20 kPa above the inlet pressure in the specimen. The volume of nZVI suspension percolated was based on the results of the batch tests (Section 2.2). The concentration of nZVI used in the tests was 4 g/L, which was the value defined in previous tests as it did not reduce the natural permeability of the soil [38]. The suspension was forced to percolate in an upward flow through the sample, and leachate was collected at the exit of each specimen.

Predetermined amounts of the contaminant solution and the soil were added and the soil was homogenized. Using this contaminated soil, the cylindrical specimens were prepared as 5 cm in diameter and 5 cm in height, with the same characteristics as natural soil. After 24 h of nZVI percolation, the specimens were sectioned into 4 equal parts of 1.25 cm (layers A through D, in relation to the nZVI inlet), and soil and leachate samples were analyzed. The remediation efficiency was calculated based on the measured Cr(VI) concentration in each layer of the specimen and the leachate as compared to the total Cr(VI) added in the specimen. Column tests under each injection pressure were conducted in triplicate to ensure repeatability of the test results.

### 2.4. Nanoscale Zero-Valent Iron (nZVI)

The nanoparticles were acquired from the NANOIRON s.r.o company (Židlochovice, Czech Republic) [39], in powder form, with contained surfactant in it, under the commercial name Nanofer Star. The nZVI consists of 65% to 85% of iron (Fe) and 20% to 30% of magnetite (Fe_3_O_4_) and iron oxide. The nanoparticles exhibit an average size of 50 nm, an average surface area of 20–25 m^2^/g. 

Following the manufacturer’s instructions, the nZVI powder was activated by using a high-speed disperser (800 W industrial blender) using a ratio of 100 g of nZVI to 400 mL of distilled water for 10 min, thus forming a suspension with 250 g/L. From the activated nZVI slurry, the necessary dilutions were carried out to yield different concentrations and then were used for the tests conducted. The activation process aimed to separate the nanoparticles that were agglomerated, due to their own reactivity, and thus ensuring the particles nanoscale.

### 2.5. Contaminant Analysis

In all experiments performed, the soil was contaminated with a standard commercial Cr(VI) 10,000 mg/L solution to result in 800 mg/kg (on dry basis) in the soil. This value corresponds to 2 times the intervention value for industrial areas according to the Brazilian Law [34]. For these values, the environmental agency requires remediation of the contaminated soil. The concentrations of contaminant in leachate and soil were determined using the USEPA 3060A and USEPA 7196A [40,41].

### 2.6. Statistical Analysis

The results were analyzed by analysis of variance (ANOVA) and Tukey’s *t*-test, with 95% confidence level (*p* value < 0.05), to compare the means between treatments, using the Statistica 5.5 software [42].

## 3. Results and Discussions

**Figure 1:**
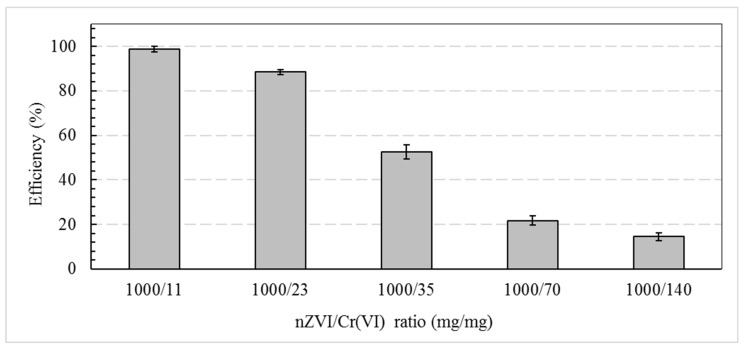
Chromium (VI) reduction efficiency in soil with different nanoscale zero Valente iron (nZVI) ratios.

The results of batch tests with the different nZVI/Cr(VI) ratios tested are shown in Figure 1. It can be observed that the reduction of the contaminant was directly related to the amount of nZVI added to the soil. At the highest nZVI ratio (1000 mg/11 mg), the reduction value reached 98%. At 43.5 (1000/23), an efficiency of 87% was achieved. Statistical analysis showed that there was a difference between the values found from a *p* value < 10^−6^, thus proving that the amount of nZVI in the soil influenced the reduction of the contaminant.

Di Palma et al. [8] used a 3 for 1 part stoichiometric ratio to remediate chromium (VI) contaminated soil (3Fe^0^ + Cr_2_O_7_^2−^ + 7H_2_O → 3Fe^2+^ + 2Cr(OH)_3_ + 8OH^−^), but reduction results between 86% and 91% were only obtained using a ratio 25 times higher than the stoichiometric ratio, demonstrating the need for an excess of reducing agent to have the contact between the contaminant and nZVI.

In Pei et al. [34], nZVI showed results close to 100% reduction of hexavalent chromium, in batch tests. However the addition of vinegar residue, which increased the amount of organic matter in the soil, helped the process of contaminant adsorption–reduction. Similar data were obtained in other studies with the addition of organic compounds, with biochar being the main one used [15,43,44]. However, the synthesis of a good support material can be costly due to the additional energy consumption, which will increase the cost of pollutant remediation. As the study soil does not present significant organic matter, the results of the reduction of the contaminant had total influence on the amount of nZVI added to the soil. 

In addition, as Cr(VI) is an anion, its sorption is favored in lower pH soils because the hydrated surface is more positively charged. That is, chromium exists in anionic HCrO_4_^−^ and CrO_4_^2−^ species, which are absorbed on adsorbents with positive charge and more favored at low pH due to protonation effect. Chromium (III) may precipitate as Cr(III) hydroxides and or Fe(III)/Cr(III) oxyhydroxides, which are incorporated into a reduced nZVI shell [17,34]. These products of the contaminant reaction with iron nanoparticles are highly stable in a pH range between 4.8 and 13.5, which fits within the natural characteristics of the soil tested in this study.

For alkaline soils, due to their high OH^−^ value, they strongly corrode the surface of iron nanoparticles, there is a deficiency of H+ to eliminate this corrosion, and the nanoparticles do not react well with chromium. For these reasons, in acidic soils, favorable conditions exist for the reduction and immobilization of the contaminant [18,45,46].

Based on the batch test results, column tests were performed to evaluate the mobility and degradation of the contaminant in the soil, simulating the field conditions. The ratio of 1000/23 was selected as it is considered adequate to reduce the contamination to the level specified in the Brazilian legislation. Using a nZVI concentration of 4 g/L, it was necessary to percolate around 1 L of suspension in each specimen to achieve the desired ratio.

Results of contaminant reduction efficiencies for each pressure used in the column tests are presented in Table 2.

The contaminant reduction efficiency values were significantly influenced by the applied pressure, even though it was much lower than the values obtained in the batch tests. This is because the homogenization of the soil contaminated the reducing agent in the batch tests, resulting in a longer contact time and a better spread of the nanoparticles in the soil, thus influencing the results obtained. The low efficiency in the column tests was also demonstrated by Vilardi et al. [19], in which a maximum efficiency between 64% and 68% was achieved, having a high influence on the contact time between the nanoparticles and the contaminant.

Analysis of variance indicates that there was a statistical difference between efficiencies as a function of pressure with a *p* factor of 0.022841. The 30 kPa and 100 kPa pressures present statistically equal values as a function of the variability between the specimens, but different from the results for the 10 kPa pressure. Thus, pressures above 30 kPa can yield good mobility of nZVI in the soil.

**Figure 2:**
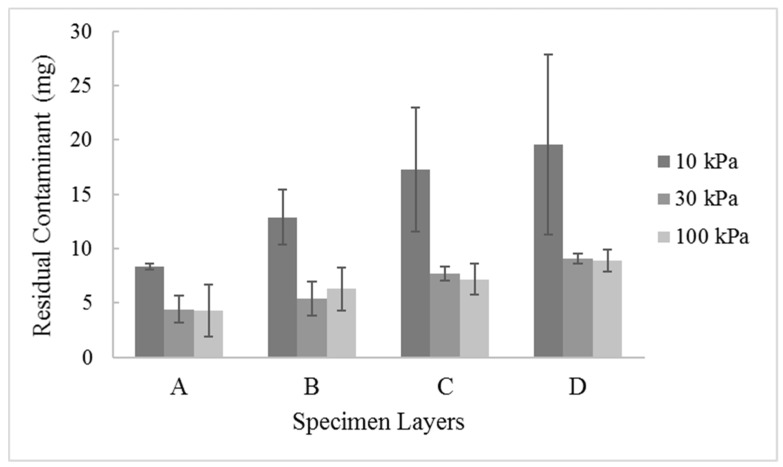
Chromium (VI) residual value in different soil layers.

Analyzing the specimens and leachate, it was observed that in the first layers of the specimen, the residual Cr(VI) contaminant values were lower when compared to the other layers. This was observed regardless of the injection pressure used. However, the residual contaminant values were lower in all layers for specimens subjected to higher pressure values (30 kPa and 100 kPa) as compared to pressure of 10 kPa, as shown in Figure 2.

Over time, nZVI reactions decrease, as reduced Cr(OH)_3_ is incorporated into the surface FeOOH layer and forms a passive layer of (CrxFe1-x)(OH)_3_ or CrxFe1-xOOH. This gradually reduces the reduction rate of Cr_2_O_7_^2−^ by Fe^0^ [17,42]. With the formation of a reduced chromium passive layer, iron nanoparticles end up losing efficiency as this layer prevents all nZVI from being oxidized and thus reducing the contaminant. In this sense, it is also justified because the use of a stoichiometric relationship does not work, since little nZVI ends up in contact with the contaminant. In addition, the formation of reduced chromium oxyhydroxides eventually causes a larger clogging of soil voids, which increasingly limits the passage of nanoparticles throughout the soil. Thus, the filtering effect of nanoparticles ends up increasing as Cr(VI) decreases.

**Figure 3:**
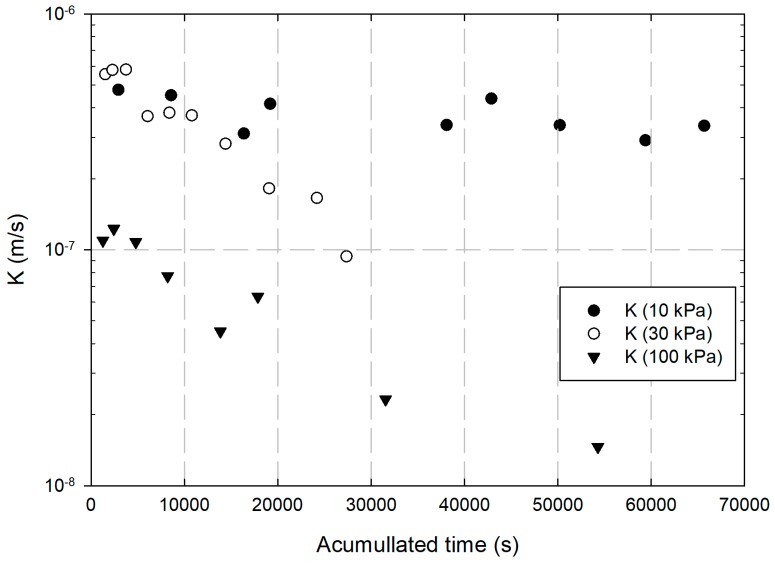
Permeability variation with elapsed time under different pressures applied.

Another problem that occurs is that with the appearance of the iron oxyhydroxide layer and an increase in filtration of nZVI, it was observed that a considerable portion of the contaminant ended up leaching from the specimens, implying a greater spread of the contamination plume if it were applied in the field. As pressure increased, a decrease in specimen permeability was also observed throughout the test, as shown in Figure 3.

For the 10 kPa pressure, there was no reduction in the permeability of the specimens. For the 30 kPa and 100 kPa pressures, a gradual reduction in permeability was observed, showing a greater filtration effect and a direct relationship with the formation of chromium (III) oxyhydroxide, helping in the clogging of the specimen. This is also explained by higher Cr(VI) reduction values for higher pressures.

## 4. Conclusions

Based on the experimental results, the following conclusions can be drawn:

1. nZVI is efficient for reducing Cr(VI) in clay soils.
2. Injection of nZVI under high pressures could lead to Cr(VI) leaching, and its mobility in soil is low due to its reaction with the contaminant.
3. Clogging of soil voids with nZVI could occur, resulting in reduced soil permeability.
4. Low contaminant reduction is due to nZVI’s poor stability and mobility and its tendency to aggregate further reduce reduction reactivity.
5. To remedy metal polluted soils, further studies are needed to improve mobility and reactivity of nZVI, especially when field application is desired.

## Figures and Tables

**Table 1:** Physical–chemical and geotechnical characteristics of the soil.

Parameter	Value
Clay (%)	68
Silt (%)	5
Sand (%)	27
Natural moisture content (%)	34
Unit weight (kN/m^3^)	16.03
Void ratio	1.24
Degree of saturation (%)	73.5
Porosity (%)	54
pH	5.4
Organic matter (%)	0.5
Cation Exchange Capacity (CEC) (cmolc/dm^3^)	8.6
Hydraulic conductivity (m/s)	1.39 × 10^−5^

**Table 2:** Soil contaminant reduction efficiency with different pressures used.

nZVI Injection Pressure (kPa)	Cr(VI) Reduction Efficiency (%)	
	Average	Standard Deviation
10	23.6	1.5
30	48.7	2.0
100	47.2	5.2
